# The evolution of parental care in insects: A test of current hypotheses

**DOI:** 10.1111/evo.12656

**Published:** 2015-04-30

**Authors:** James D J Gilbert, Andrea Manica

**Affiliations:** 1 Department of Evolution, Behaviour and Environment, University of SussexBrighton, BN1 9QG, United Kingdom; 2 School of Biological, Biomedical and Environmental Science, University of HullHull, HU6 7RX, United Kingdom; 4 Department of Zoology, University of CambridgeCambridge, CB2 3EJ, United Kingdom

**Keywords:** Arthropods, comparative method, exclusive paternal care, life-history evolution

## Abstract

Which sex should care for offspring is a fundamental question in evolution. Invertebrates, and insects in particular, show some of the most diverse kinds of parental care of all animals, but to date there has been no broad comparative study of the evolution of parental care in this group. Here, we test existing hypotheses of insect parental care evolution using a literature-compiled phylogeny of over 2000 species. To address substantial uncertainty in the insect phylogeny, we use a brute force approach based on multiple random resolutions of uncertain nodes. The main transitions were between no care (the probable ancestral state) and female care. Male care evolved exclusively from no care, supporting models where mating opportunity costs for caring males are reduced—for example, by caring for multiple broods—but rejecting the “enhanced fecundity” hypothesis that male care is favored because it allows females to avoid care costs. Biparental care largely arose by males joining caring females, and was more labile in Holometabola than in Hemimetabola. Insect care evolution most closely resembled amphibian care in general trajectory. Integrating these findings with the wealth of life history and ecological data in insects will allow testing of a rich vein of existing hypotheses.

The question of which sex should evolve to care for offspring has received much theoretical attention. The caring sex is thought to be determined by several nonexclusive factors, for example, mate competition (e.g., Trivers 1972; Kokko and Jennions 2008), territoriality (Williams 1975), physiology of gamete release (Dawkins and Carlisle 1976; Gross and Shine 1981); cuckoldry risk (Maynard Smith 1977; Queller 1997), and sex-specific life history (Klug et al. 2013a,b). To date, though, comparative-phylogenetic studies of transitions in the caring sex have been restricted to vertebrates (Szekely and Reynolds 1995; Beck 1998; Goodwin et al. 1998; Burley and Johnson 2002; Reynolds et al. 2002; Mank et al. 2005, although see Kutschera and Wirtz 2001), despite calls for similar studies of invertebrates (Reynolds et al. 2002). Insects constitute one invertebrate group where parental care is extremely diverse. Parental care, where present, ranges from temporary egg-guarding to feeding of offspring by both parents up to adulthood (Eickwort 1981; Hinton 1981; Tallamy and Wood 1986; Choe and Crespi 1997; Costa 2006). Care can be by females, males, or both, sometimes with multiple such strategies within families (Halffter 1997), genera (Gilbert et al. 2010), and even species (Beal and Tallamy 2006). Insects contain some of the best-characterized examples of exclusive male care, the rarest form of care in nature. Yet studies of evolution of the caring sex in insects are largely limited to detailed but nevertheless qualitative accounts (e.g., Tallamy and Wood 1986; Tallamy and Schaefer 1997; Tallamy 2001; Trumbo 2013; Wong et al. 2013, but see Manica and Johnstone 2004; Field 2005).

Female care is widespread in groups related to the Hexapoda such as the Arachnida, Isopoda, Chilopoda, Diplopoda, and Onychophora (Zeh and Smith 1985). The earliest insects lost care when they evolved desiccation-proof eggs, and ovipositors to hide them (Zeh et al. 1989). Insects that re-evolved care were typically those with unusually harsh or rich environments; stable, structured environments; or high levels of predation (Wilson 1975), although these four well-known “prime movers” of parental care may possibly be described more parsimoniously as a function of species’ feeding ecology—the “resource hypothesis” (Tallamy and Wood 1986). Additionally, female care was promoted in those species that secondarily lost their ovipositor or reduced investment in the protective egg wall (Zeh et al. 1989; Smith 1997). Female care may have also been favored by semelparity (Tallamy and Brown 1999, but see Trumbo 2013). Internal fertilization (such as in insects) enables males to desert offspring earlier than females (Dawkins and Carlisle 1976), physically dissociates males from embryos (Williams 1975), and reduces paternity certainty, selecting against male care (Trivers 1972; Queller 1997; Kokko and Jennions 2003). More generally, male care is disfavored whenever males compete for females (whether pre- or postcopulation)—a common situation in many populations with anisogamy and 1:1 sex ratios, but especially in insects, where females store sperm in spermathecae, leading to widespread sperm competition (Simmons 2001). In this scenario, increased variance in male success means some males sire zero offspring and their parental care decisions are invisible to selection, whereas other males sire offspring by many females and therefore statistically stand to gain by deserting to remate—selecting against male care (Queller 1997; Kokko and Jennions 2008).

Male insects therefore require a very good reason to perform parental care. Males are predicted to care alongside females if they have low chances of breeding again (e.g., Shachak 1980), or if biparental cooperation is highly beneficial, such as in species that use, defend, or entirely live within “bonanza”-style resources (Trumbo 1994; Nalepa 1988; reviewed in Zeh and Smith 1985)—but are nevertheless expected to be sensitive to further mating opportunities (Eggert and Sakaluk 1995). Exclusive male care is facilitated if males mate-guard females until oviposition, or defend breeding territories, both of which reassociate males and eggs (Zeh and Smith 1985).

Hypotheses concerning the evolution of exclusive male care can be divided according to the direction in which male care is predicted to evolve. First, male care may evolve directly from no care; second, male care may evolve out of female care—either directly or via a biparental intermediate. The first case arises where costs of care in terms of missed mating opportunities are reduced for males, for example, if males can care for many females’ broods at once (Williams 1975; Zeh and Smith 1985)—dubbed the “overlapping broods hypothesis” (Manica and Johnstone 2004). Selection for male care is enhanced if females later evolve to prefer mating with caring males (Tallamy 2000).

In the second case, male care could evolve from female- or biparental care if the cost of investing in care is greater to females than to males (Maynard Smith 1977), selecting for females to leave offspring in the sole care of the male. Initially proposed for birds and fish (Emlen 1973; Graul 1973; Nethersole-Thompson 1973; Sargent and Gross 1993, see Kokko and Jennions 2008), this hypothesis was co-opted for invertebrates as the “enhanced fecundity” hypothesis (Tallamy 1994, 2000). To evolve care, though, males must directly benefit by caring (Ridley 1978). Proponents of this idea have argued that male care can be seen as a “gift” to the female, analogous to a nuptial gift, which makes the male attractive (Tallamy 1994), that is, female preference for caring males can provide a benefit to males who take over parental duties from females. On one level this begs the question, because male care must have preceded female preference for caring males. However, it is possible that males can also benefit directly by caring, as nuptial gifts can increase the male's number of progeny by increasing female fecundity in the current brood (Boggs 1990; Gwynne 2008, but see Vahed 1998). Thus, in principle it is possible that males may benefit, even in the current brood, by freeing females from costs of care they would otherwise incur. This “gift of cost-free care” (Tallamy 2000) could potentially increase the current brood size compared to what the female could lay if she were caring—helping to select against the male deserting to remate. For this mechanism to work, though, females must initially perform parental care, otherwise males cannot benefit by freeing them from its energetic cost. Thus, this mechanism requires that male care evolves out of female care, or a biparental intermediate.

Here, we present the first attempt to reconstruct transitions in the caring sex across the insect phylogeny, testing predictions arising from hypotheses discussed above. Note that we are not explicitly testing mechanisms of insect parental care evolution (see Wong et al. 2013 for a recent review), but instead the predicted direction in which parental care will evolve. Because internal fertilization and sperm competition select against male care, we predict the most common transitions are between no care and female care, and biparental care evolved from female care, not from male care. For male care, we test between two alternative hypotheses: “N2M,” male care arose from no care (predicted by the overlapping broods hypothesis), or “F2M,” male care arose via a female or biparental intermediate (predicted by the enhanced fecundity hypothesis).

For our analyses, we broke the insect phylogeny up into Holometabola (1363 species) and Hemimetabola (651 species). This makes sense because offspring of these two groups are fundamentally different; hemimetabolous nymphs are typically mobile and self-sufficient, with similar bauplan and lifestyle to adults; in contrast, holometabolous larvae are often immobile and undergo metamorphosis before adulthood, usually involving major shifts in ecology. Furthermore, there are no known exclusive male carers in the Holometabola, suggesting patterns of parental care evolution may differ between the two.

## Methods

We gathered literature data on insect parental care (sex of the care-giver), and matched them up to a phylogeny constructed from published sources. We then used a combination of phylogenetic methods to infer the prevailing directions of parental care evolution in insects.

### DEFINITION OF PARENTAL CARE

We conducted literature searches using ISI Web of Knowledge (http://wok.mimas.ac.uk) and Google Scholar (http://scholar.google.com). We coded the caregiving sex as a factor with four discrete, unordered levels: “no care,” “female care,” “male care,” and “biparental care.” Parental care is traditionally defined as costly behavior by parents that increases fitness of offspring (Clutton-Brock 1991). Subsequently, this definition has been expanded to “parental behavior that arose and/or is maintained for the purpose of increasing offspring fitness” (Smiseth et al. 2012). Broadly following Reynolds et al. (2002), we defined insect parental care as postovipositional guarding, carrying or cleaning of offspring; preoviposition or postoviposition provisioning; ovoviviparity; or viviparity—but with the following caveats. Care by males was only included when it was not clearly a prerequisite for mating. We did not include, for example, nuptial gifts that may or may not be used by females to nourish offspring (e.g., in katydids; Gwynne 1988, but see Vahed et al. 2014), or male nest-building if this functioned as a prerequisite for securing mates (e.g., Halffter 1997). Male *Trypoxylon* wasps that guard larvae while females forage, for instance, were considered to exhibit care, making them biparental (Coville and Coville 1980; Coville and Griswold 1984), whereas most male Scarabaeine beetles, who typically do not participate after copulation, were not (discussed in Halffter 1997). Cases described as male care where males guard eggs inside a territory as part of a mating system of resource-defense polygyny, for example, in some thrips (e.g., *Hoplothrips* spp.; Crespi 1986, 1988), were treated as male care (see, e.g., Tallamy 2001; Costa 2006); however, guarding of ovipositing mates from other males, for example, in the Odonata, was not. Species with workers, such as ants and termites, were excluded, because (1) alloparental care is likely to be subject to an array of different selection pressures from those favoring care by the parent, and (2) the presence of workers per se is likely to alter selection pressures acting upon parents, complicating the picture. For consistency, and in line with the majority of the literature, cases where eggs were coated with secretions, frass, or soil were not classified as parental care— even in the few cases where they are described as such (e.g., Schatz 1994).

### ABSENCE OF PARENTAL CARE

In general, the absence of parental care was almost never explicitly stated except, rarely, when contrasted against caring species (e.g., Tallamy and Denno 1981; Hanelová and Vilímová 2013). Rather, we examined descriptions of species’ behavior up to and following oviposition, and denoted “no care” if no mention was made of our key criteria (see above). We also accepted statements such as “no known members of group X perform parental care,” where explicitly stated and where no contradictory evidence could be found, and applied these across the group in question.

### TAXON SAMPLING

Taxon sampling is a critical issue with such a diverse group. Even though we gathered what might be considered a large sample of over 2000 species, this constitutes barely 0.2% of the approximately 1 million described species of insect (Grimaldi and Engel 2005). We sampled relatively thinly in groups where no variation in the caregiving sex is known (e.g., Odonata [no care], Embiidina [female care], Hemiptera:Belostomatidae [male care]). We concentrated our effort in groups with variation in the caring sex. In these groups, we identified caregiving species from literature studies and then used published phylogenies to identify lineages closely related to these caregiving species or, if unavailable, to their lowest identifiable containing taxon (their subtribe, tribe, etc.). We then searched for biological descriptions of the reproductive behavior of these taxa.

### THE PHYLOGENY

To assemble the insect phylogeny, we collected published phylogenies encompassing species of interest (see Table S1), and joined them together manually in jigsaw fashion (see, e.g., Webb and Donoghue 2005; Wiens et al. 2006). We favored current reviews summarizing recent phylogenetic work, principally the Tree of Life (http://tolweb.org/), a conservative synthesis of current knowledge. Because none of the phylogenies was based upon reproductive characters, errors in these phylogenies were assumed to be random with respect to transitions in the caring sex (following Goodwin et al. 1998). Branch lengths were incompatible among phylogenies, so were arbitrarily scaled according to node depth, following Grafen (1989).

Many taxa for which data existed had not to our knowledge been formally placed on a phylogeny. We used accepted taxonomic classification to place these taxa to the smallest known level onto the phylogeny. This created polytomies (multiple nodes) at nodes where these unknown species were added. The resulting tree had 2013 species and 1253 nodes, spanning 24 out of an estimated 30 insect orders. A total of 290 nodes (23.1%) were polytomous, that is, with three or more daughter nodes, 146 nodes (11.7%) had four or mode daughter nodes, and 90 (7.1%) had five or more daughter nodes. Following Reynolds et al. (2002), we partially addressed the resulting phylogenetic uncertainty using multiple alternative topologies—in our case created by random resolutions of these polytomies across the tree (see below for details). This phylogeny was then broken up into Holometabola (1363 tips) and Hemimetabola (651 tips) with analyses performed separately on each clade.

### EVOLUTIONARY HYPOTHESES OF THE CARING SEX IN THE HOLOMETABOLA

There are no known exclusive male carers in the Holometabola, so transitions to and from male care are impossible unless we posit hypothetical states. In the interests of parsimony, we removed this possibility from our models, while recognizing this as a conscious choice (following, e.g., Beaulieu and Donoghue 2013).

Using the program BayesTraits version 2.0 (Pagel and Meade 2011) in MultiState mode, we fitted four broad evolutionary models of parental care evolution. The first two models examined our predictions that transitions between no care and female care would predominate among insects, and that biparental care would arise out of female care. The FULL model assumed transitions among parental care states were unrestricted. The DISCRETE model assumed only one sex could change care state at any one time (i.e., restricting to zero those transition rates that involve two changes, such as no care to biparental care). The remaining two models additionally tested whether transitions and reversals occurred with similar frequencies. The FULL.REV model was similar to the FULL model but with the evolutionary rates for the backwards and forwards transitions restricted to be equal. The DISCRETE.REV model was similar to the DISCRETE model but again with backwards and forwards transitions restricted to be equal.

### EVOLUTIONARY HYPOTHESES OF THE CARING SEX IN THE HEMIMETABOLA

In the Hemimetabola, all four possible care states exist. To test the hypotheses outlined above regarding parental care evolution in the Hemimetabola, we again fitted the FULL and DISCRETE models, but multiple times incorporating several additional evolutionary assumptions in different combinations.

### EVOLUTION OF MALE CARE

1. F2M (female or biparental care precedes male care, for example, Enhanced Fecundity model): transitions from no care to male care restricted to zero such that male care arises only from female care or biparental care.
2. N2M (male care arises from no care, for example, Overlapping Broods model): transitions from female care to male care restricted to zero. (Note that this model does not make predictions about transitions between male care and biparental care; note also that this is consistent with an unmodified DISCRETE model above, so we removed redundant combinations as appropriate).
3. NR, unrestricted model.

### EVOLUTION OF BIPARENTAL CARE

1. MALEJOINS, biparental care arises only via males joining females: transitions from male care to biparental care restricted to zero.
2. NR, unrestricted model.

**Figure 1:**
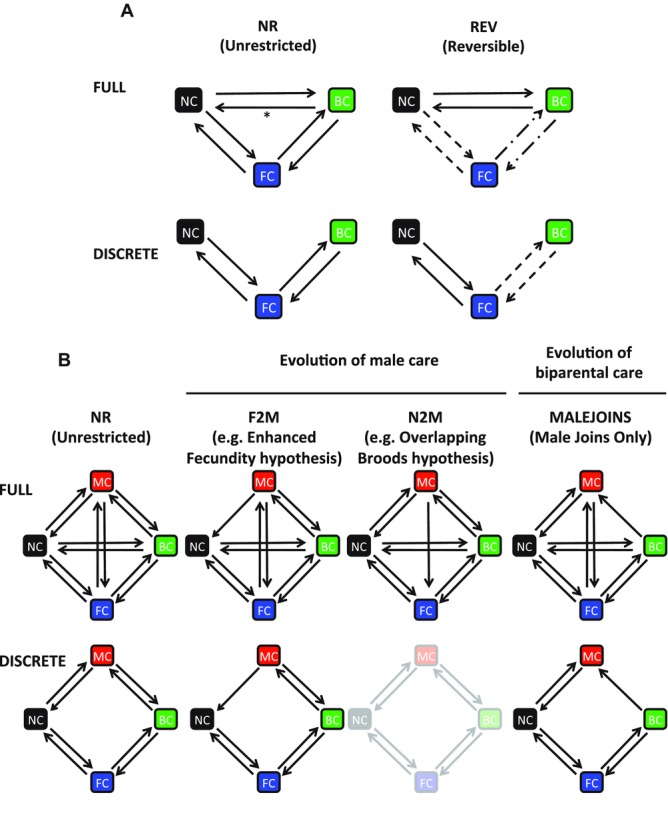
Schematic representation of models tested in (A) Holometabola and (B) Hemimetabola datasets. For the reversible models in the Holometabola, transition rates represented by similar line types (solid, dashed, dot-dashed) are constrained to be equal. For Hemimetabola, the DISCRETE.NR and DISCRETE.N2M models are redundant, so one is arbitrarily grayed out. Asterisk (*) shows the rate that was set to zero in the modified FULL model (see Results for details).

To test our key hypotheses, we evaluated models comprising all permutations of these evolutionary assumptions—for example, the DISCRETE.F2M.MALEJOINS model assumes (1) only one sex can change care state at one time, (2) transitions from no care to male care are restricted to zero, and (3) transitions from male care to biparental care are restricted to zero. The full sets of tested models for both datasets are shown in schematic form in Figure1.

### ESTIMATING EVOLUTIONARY TRANSITION RATES

We analyzed the models outlined above in two ways. In the first approach, we treated the tree (with polytomies representing uncertainty) as the one true tree, and evaluated the model set and the transition rate parameters assuming this single tree was correct—thus obtaining an estimate of the best model and the most likely parameter values on one uncertain tree, but without an estimate of uncertainty due to phylogeny. To be able to incorporate uncertainty due to the phylogeny into our confidence intervals, in the second approach we evaluated the model set across a sample of 1000 trees generated by randomly resolving all polytomies in the original tree.

### FINDING THE BEST MODEL FOR THE ORIGINAL TREE

To analyze the models using only the single original polytomous tree, we used a reversible-jump Markov-Chain Monte-Carlo (RJMCMC) approach implemented in BayesTraits version 2.0 (Pagel and Meade 2011). The RJMCMC algorithm evaluates the entire universe of possible evolutionary models simultaneously, by walking through a landscape represented by all possible models (and normally also all possible trees, although in our case the set of possible trees includes only one tree). The algorithm visits each possible model in proportion to its likelihood given the data and tree, thus providing us with a posterior sample of the most likely models. We are then able to evaluate the hypotheses we are concerned about by assessing support for those models in the posterior sample that correspond to our hypotheses.

In the case of the Hemimetabola, a “model” is described by a string of 12 transition parameters (representing the 12 possible transitions among four care states), collectively describing which rates are similar, which are different, and which are zero. Each rate takes one of three states: (1) set to Z, that is, zero, (2) set as equivalent to another rate such that one parameter parsimoniously describes both rates, or (3) assuming a value independent of the other rates. For example, one model might be represented by the string “aaaaaabacaZZ,” indicating that, in this model, most rate parameters are equivalent and are described by one value (“a”), except parameters 7 and 9 that take independent values (“b” and “c,” respectively), whereas parameters 11 and 12 are both set to zero. Testing our F2M assumption (e.g.), then, requires us to evaluate the support for the set of models where parameter 12 (rate of transitions from no care to male care) is set to Z.

Support for each model can be assessed by its Bayes factor (BF), the ratio of the model's posterior to prior odds; a BF below 1 we treated as evidence against the model, 1–3 as positive evidence in favor of the model, 3–10 as substantial, 10–30 as strong evidence, 30–100 as very strong evidence, and >100 as decisive (Kass and Raftery 1995, see Currie et al. 2010). Posterior odds are calculated as the frequency of a given model in the posterior RJMCMC sample. In contrast, prior odds are calculated as the frequency that a given model appears in the universe of possible models.

To determine how many models constitute the universe of possible models, we followed the calculations of Currie et al. (2010). The bell number is the total number of possible ways of combining *n* objects (12 rate parameters for the Hemimetabola) into any number of different classes (i.e., different independent values in the model string). For 12 nonzero parameters, the bell number is 4,213,597. However, in our case one or more parameters can additionally be restricted to zero, expanding the set of possible models. For 12 parameters of which exactly one is restricted to zero, the number of possible models equals the bell number for the remaining 11 nonzero parameters, multiplied by the 12 possible ways of having one single zero parameter. For more than 1 and up to 11 zero values, Table S2A shows calculations of the numbers of possible models (reproduced from Currie et al. 2010). The sum of these numbers plus the bell number for 12 nonzero parameters gives the total number of possible models for Hemimetabola, which equals 27,644,436 (Table S2A and Currie et al. 2010). The total number of possible models for the Holometabola, with six parameters, is much lower at 876 (Table S2B).

As an example from the Hemimetabola, the prior odds of our F2M model (described above) equals the number of possible models where parameter 12 (the no care to male care transition) is set to zero divided by the number of possible models where this transition is not set to zero (equal to 678,570/(27,644,436 – 678,570) = 0.02516).

To propose values for each nonzero transition rate parameter, we used a hyperprior to provide the parameters of an exponential prior distribution, drawn from a uniform distribution with range 0.1–5; this interval was chosen based on an initial run of the model with unrestricted rates in maximum likelihood (ML) mode. An exponential distribution was used instead of the more usual gamma distribution because some estimated rates approached zero (see Results), which prevented model convergence using a gamma hyperprior.

We ran five chains for each RJMCMC model and used the harmonic mean likelihood to check for convergence. Each model was run for 50,100,000 iterations with sampling period 5000 to avoid autocorrelation, discarding the first 100,000 as burn-in.

### INCORPORATING UNCERTAINTY DUE TO PHYLOGENY

In any analysis of trait evolution, there is uncertainty associated with both the parameters and tree. In this case, the tree is especially poorly resolved, having been assembled from disparate published trees, with arbitrary branch lengths and many taxa added simply by classification. Such a structure is inescapable given the diversity of insects and number of unplaced taxa. Our interest here is to provide a broad overview of parental care evolution and test its robustness in the face of plausible uncertainty in the reconstruction of the tree.

Although the single-tree RJMCMC approach allows us to estimate uncertainty in parameters given the data and given a single assumed true phylogeny, it does not take into account uncertainty in the tree. To assess the robustness of our conclusions to plausible variation in the phylogeny, we created a sample of 1000 trees with random resolutions of all polytomies (i.e., generating random hierarchical relationships among species whose affinities were uncertain). We did not distinguish between polytomies identified in the literature and polytomies created by adding unplaced taxa. After resolving polytomies, for each tree we recomputed branch lengths according to node depth, multiplied each branch by a random scaling factor drawn from a normal distribution with mean 1 and SD 0.2, and then rescaled each tree to a uniform total node depth of 1 using the rescaleTree() function in the geiger package (Harmon et al. 2008).

As we had no measure of tree likelihood, all trees were equally weighted. Under these premises, a Bayesian approach was inappropriate because the MCMC algorithm would gravitate toward trees that happened to fit the data more parsimoniously (A. Meade, pers. comm.). Instead, we used BayesTraits running in ML mode to estimate rate parameters for all candidate models on each tree, and assessed hypotheses based on the resulting 1000 within-tree AIC tables. Within each AIC table, all models were compared simultaneously on the basis of ΔAIC to the top model (favoring models with ΔAIC < 2; Burnham and Anderson 2002). We also converted ΔAIC to Akaike weights, providing an estimate of the conditional probability for each model given the data and set of candidate models—and then used the evidence ratio, the ratio of Akaike weights, to conduct comparisons between competing model sets of interest.

The rate parameters estimated by RJMCMC and ML methods were formally incomparable owing to the rescaling of the trees between methods, so we instead compared the relative performance of models within analyses, noting any discrepancies.

### NUMBERS OF TRANSITIONS

We used stochastic character mapping to provide estimates of the historical numbers of transitions of each type within each tree, and summarized these estimates across trees. We used the diversitree package (FitzJohn 2012 and R. Fitzjohn, pers. comm.) to simulate ancestral states conditional on the ML parameters estimated by BayesTraits. For each of the 1000 trees, we simulated 100 stochastic character histories based upon the FULL model in the ML analysis.

## Results

**Figure 2:**
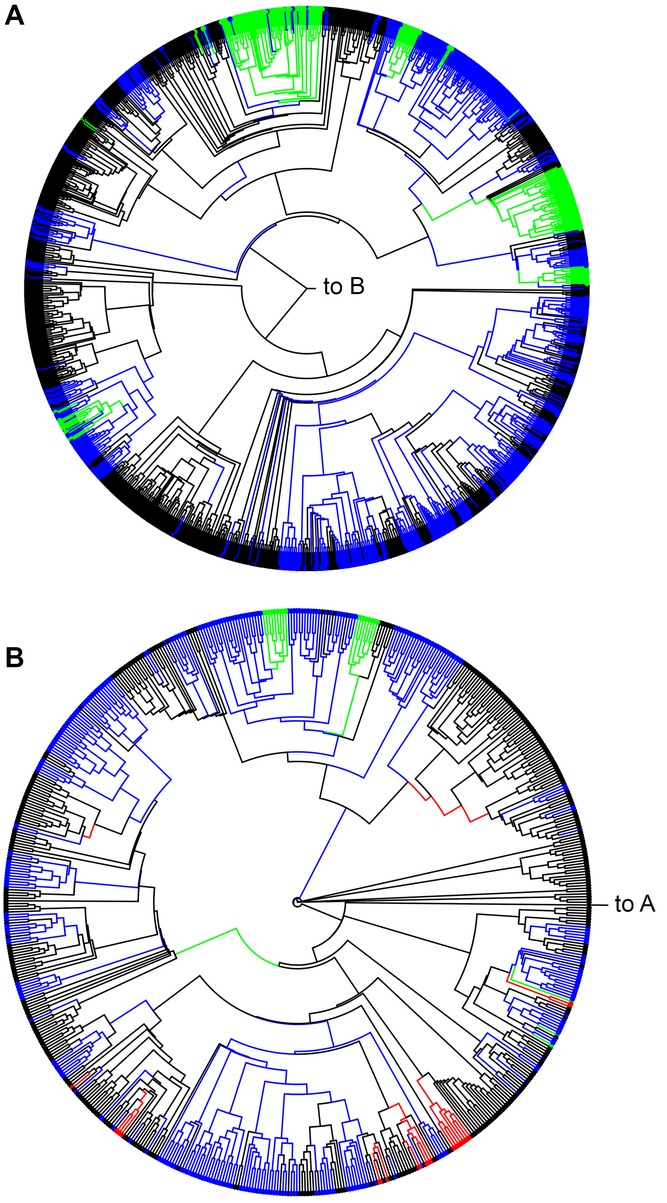
One of 1000 randomly resolved phylogenies of parental care evolution in 2013 insect species, with ancestral parental care states added using stochastic character mapping: (A) Holometabola, (B) Hemimetabola. Labels indicate the point where the two phylogenies join. Color key: black, no care; blue, female care; red, male care; green, biparental care.

One resolution of the complete phylogeny is shown in Figure2, with extant and estimated ancestral parental care states shown; a higher resolution phylogeny is available as Figure S1. Data are given in Table S3.

### EVOLUTION OF THE CARING SEX IN THE HOLOMETABOLA

**Figure 3:**
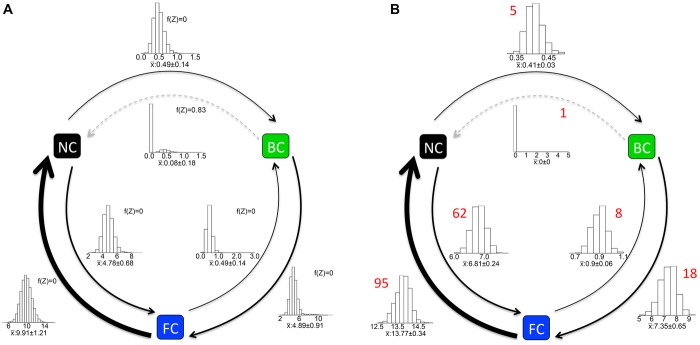
Transition rates in the Holometabola estimated from (A) the RJMCMC model of the single, polytomous tree and from (B) 1000 unrestricted (i.e. FULL.NR) ML models of randomly resolved trees. Arrow width is proportional to transition rate; gray dashed lines indicate rates where SD of the rate distribution overlapped zero. In (A), histograms show the posterior distribution of rates across all five RJMCMC chains (*n* = 500,900); the median ± SD is given separately along with the frequency the rate was set to Z. In (B), histograms show distributions of the ML values for each rate across 1000 random resolutions of the parent tree with median ± SD given separately; figures above graphs indicate median transition counts per tree, estimated by stochastic character mapping using an unrestricted (i.e. FULL.NR) model.

Transition rates from the Bayesian analysis of the single unresolved tree are shown in Figure3A, whereas results from the ML analyses, along with estimated transition counts, are shown in Figure3B. In the Holometabola, in both the Bayesian and ML analyses, transitions between no care and female care were most likely. Biparental care evolved rarely, but arose both from no care and from female care with approximately equal probability. Transitions from biparental care to no care were estimated at zero. Once evolved, biparental care tended to decay into female care rather than back to no care.

In the RJMCMC analysis, neither FULL.NR nor DISCRETE.NR models received positive support (BF ∼0.67 and ∼0, respectively; Table 1A). This was because biparental care to no care transitions were overwhelmingly set to Z, whereas no care to biparental care transitions were not. Accordingly, a modified FULL model in which transitions from biparental care to no care were restricted to zero received very strong support (BF ∼78; Table 1A). Thus, transitions directly from no care to biparental care were strongly supported, although occurring at a low rate.

**Table 1:** Table of Bayes factors for RJMCMC analyses of (A) Holometabola and (B) Hemimetabola

	RJMCMC run									
(A)	1		2		3		4		5	
HML	−615.07		−614.31		−614.71		−614.31		−614.11	
	*N*	BF	*N*	BF	*N*	BF	*N*	BF	*N*	BF
full.modified*	**83,310**	**78.25**	**83,407**	**78.80**	**83,159**	**77.42**	**83,926**	**78.18**	**83,420**	**77.87**
full.nr	16,865	0.67	16,765	0.67	17,017	0.68	16,875	0.67	16,935	0.67
discrete.nr	76	0.00	98	0.06	93	0.05	83	0.05	78	0.05
full.rev	0	0.00	0	0.00	0	0.00	0	0.00	0	0.00
discrete.rev	0	0.00	0	0.00	0	0.00	0	0.00	0	0.00

(B)										
HML	−324.77		−324.43		−324.42		−324.60		−324.55	
	*N*	BF	*N*	BF	*N*	BF	*N*	BF	*N*	BF
**discrete.mj**	**268**	**84.55**	**265**	**83.60**	**256**	**80.75**	**220**	**69.37**	**247**	**77.91**
**full.n2m.mj**	**17,769**	**51.18**	**18,640**	**54.26**	**18,548**	**53.93**	**16,354**	**46.31**	**18,412**	**53.45**
**discrete.nr**	**558**	**37.40**	**550**	**36.86**	**570**	**38.20**	**495**	**33.15**	**567**	**38.00**
**full.mj**	**46,118**	**33.90**	**44,219**	**31.40**	**44,191**	**31.37**	**49,872**	**39.39**	**43,941**	**31.05**
**full.n2m**	**38,479**	**24.78**	**40,369**	**26.82**	**40,195**	**26.63**	**35,783**	**22.08**	**40,048**	**26.47**
full.f2m.mj	16	0.04	13	0.03	21	0.05	17	0.04	19	0.05
full.f2m	42	0.02	31	0.01	40	0.01	36	0.01	39	0.02
full.nr	67	0.00	80	0.00	85	0.01	90	0.01	88	0.00
discrete.f2m	0	0.00	0	0.00	0	0.00	0	0.00	0	0.00
discrete.f2m.mj	0	0.00	0	0.00	0	0.00	0	0.00	0	0.00

Key: HML = Harmonic mean likelihood; *N* = frequency of appearance in model chain (out of 100,180 sampled iterations); BF = Bayes factor (see text for discussion); nr = not restricted; mj = MALEJOINS model; n2m = N2M model; f2m = F2M model. Models with at least positive evidence in their favor (BF > 1) are given in bold. *Modified full model in which transitions from biparental care to no care are set to Z (zero).

Similarly, FULL.NR was preferred over DISCRETE.NR in the ML analysis across all 1000 permutations of the phylogeny (median ΔAIC 8.95, median Akaike weight 0.99, median evidence ratio 87.78; Table 2A), but the modified FULL model was always preferred over both of these models (median ΔAIC 2.00, median Akaike weight 0.73, median evidence ratio 2.68; Table 2B). “Reversible” models, with forward and backward rates constrained to be equal, were never visited by the RJMCMC algorithm; neither were they preferred by ML analyses (FULL.REV and DISCRETE.REV models, Akaike weight never > 0.001, median evidence ratio always < 10^−8^).

**Table 2:** Summary of AIC tables for ML analyses across 1000 randomly resolved trees for (A) Holometabola, (B) Holometabola, including the “full.modified” model*, and (C) Hemimetabola

(A)	AIC	ΔAIC	ΔAIC min	ΔAIC max	*F*_intop_	*F*_top_	W_Ak_	W_Ak_ min	W_Ak_ max
full.nr	1199.78	0.00	0.00	0.00	1000	1000	0.99	0.96	1
discrete.nr	1208.93	8.95	6.57	14.27	0	0	0.01	0.00	0.04
full.rev	1238.63	38.94	25.76	49.15	0	0	0.00	0.00	0.00
discrete.rev	1240.63	40.91	28.92	50.80	0	0	0.00	0.00	0.00
(B)									
full.modified*	1197.78	0.00	0.00	0.00	1000	1000	0.73	0.72	0.73
full.nr	1199.78	2.00	2.00	2.00	0	0	0.27	0.27	0.27
discrete.nr	1208.93	10.95	8.57	16.27	0	0	0.00	0.00	0.01
full.rev	1234.63	36.94	23.76	47.15	0	0	0.00	0.00	0.00
discrete.rev	1238.63	40.91	28.92	50.80	0	0	0.00	0.00	0.00
(C)									
full.n2m	652.08	0.00	0.00	3.40	985	404	0.40	0.10	0.65
full.n2m.mj	652.29	0.00	0.00	3.40	979	596	0.40	0.10	0.65
full.nr	655.93	3.99	0.00	6.73	14	1	0.06	0.01	0.24
full.mj	656.07	3.97	0.03	7.04	11	0	0.06	0.01	0.23
discrete.nr	656.97	4.84	1.43	12.08	4	0	0.04	0.00	0.16
discrete.mj	656.97	4.84	1.32	12.08	4	0	0.04	0.00	0.17
full.f2m.mj	665.80	13.87	4.25	23.84	0	0	0.00	0.00	0.03
full.f2m	665.82	13.91	4.25	23.84	0	0	0.00	0.00	0.03
discrete.f2m	674.80	23.32	12.77	60.04	0	0	0.00	0.00	0.00
discrete.f2m.mj	676.41	23.77	12.78	60.04	0	0	0.00	0.00	0.00

Key: nr = not restricted; mj = MALEJOINS model; n2m = N2M model; f2m = F2M model; *F*_intop_ = frequency that model appears among top models for tree in question; *F*_top_ = frequency that model is the top model for tree in question; W_Ak_ = Akaike weight. *Modified full model in which transitions from biparental care to no care are set to Z (zero).

Simulated numbers of each transition from stochastic character mapping are given in Figure3B. Across trees there were more transitions from no care to female care than in the reverse direction. Biparental care evolved from female care and no care approximately the same low number of times, but thereafter, biparental care evolved into female care more times than it reverted to no care.

### EVOLUTION OF THE CARING SEX IN THE HEMIMETABOLA

**Figure 4:**
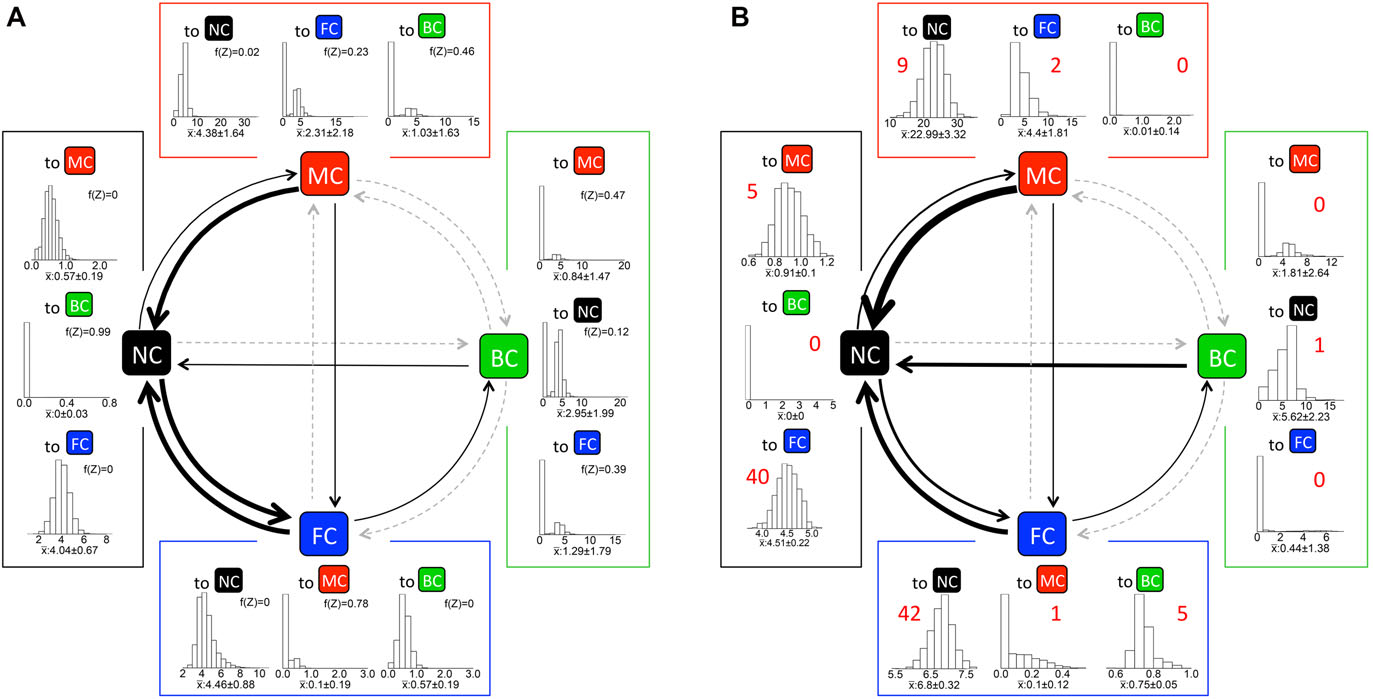
Transition rates in the Hemimetabola estimated from (A) the RJMCMC model of the single, polytomous tree and from (B) 1000 unrestricted (i.e. FULL.NR) ML models of randomly resolved trees. Arrow width is proportional to transition rate; gray dashed lines indicate rates where SD of the rate distribution overlapped zero. In (A), histograms show the posterior distribution of rates across all five RJMCMC chains (*n* = 500,900); the median ± SD is given separately along with the frequency the rate was set to Z. In (B), histograms show distributions of the ML values for each rate across 1000 random resolutions of the parent tree with median ± SD given separately; figures above graphs indicate median transition counts per tree, estimated by stochastic character mapping using an unrestricted (i.e. FULL.NR) model.

Posterior distributions of transition rates from the Bayesian analysis of the single unresolved tree are shown in Figure4A. In the Hemimetabola, transitions between no care and female care were again most likely, with female care additionally evolving further to biparental care. No care and male care also exchanged at rates substantially above zero. Between male care and biparental care, transitions were very rare. Biparental care in general was stable, decaying only rarely. Transitions from female care to male care, and from no care to biparental care, were statistically zero.

In the RJMCMC analysis, the model with the highest support was DISCRETE.MALEJOINS (BF ∼80; Table 1B), followed by FULL.N2M.MALEJOINS (BF ∼50) and DISCRETE.NR (BF ∼37). Thus, the best models, with very strong support, incorporated both N2M and MALEJOINS assumptions but rejected transitions between male care and female care. No models incorporating the F2M assumption had BFs above 0.05, and we accordingly rejected them.

In contrast, the ML analyses preferred FULL-type over DISCRETE-type models (Fig.4B): 964 of 1000 random resolutions of the parent tree gave the same two equivalent top models (FULL.N2M and FULL.N2M.MALEJOINS; median ΔAIC between these models = 0.037, median ΔAIC to next best model = 3.77; Table 2C). Within the ML analyses for each tree, N2M models (including DISCRETE models, which incorporate the N2M assumption) had a median Akaike weight of 87.0% (78.6% excluding DISCRETE models), whereas F2M models had a median Akaike weight of 0.002% (comparing N2M vs. F2M models, median evidence ratio > 1000), strongly suggesting that male care has tended to arise out of no care rather than out of female or biparental care. MALEJOINS models had a median Akaike weight of 49.6%, indicating inconclusive support for the hypothesis that biparental care arises predominantly by males joining females.

## Discussion

The evolutionary transitions we have identified across the insect phylogeny largely support the existing view of insect care evolution. From an initial scenario of no care, ubiquitous in basal insect groups and predominant in insects (Zeh et al. 1989), descendant lineages evolved down three routes: (1) no care evolved to female care, supporting the prediction about the prevalence of this transition, and in some cases further to biparental care (e.g., Zeh and Smith 1985), providing qualified support for the MALEJOINS prediction, discussed below; (2) in the Hemimetabola, no care evolved to male care (Zeh and Smith 1985), supporting the N2M but not the F2M prediction, and (3) in the Holometabola, no care evolved directly to biparental care (e.g., in burying beetles, *Nicrophorus* spp.; Eggert and Muller 1997). Female care was labile, but biparental care showed different patterns depending on taxonomy: in the Holometabola biparental care was also labile, decaying to female care, but in the Hemimetabola it was more stable.

The current uncertainty in evolutionary relationships among insects appeared strongly in our analyses. This was evidenced by a marked widening of confidence intervals and an increased number of possible transitions in the ML analysis of 1000 randomly resolved dichotomous trees, compared to the Bayesian analysis of one unresolved tree (Figs. 3 and 4). For example, at any polytomy where the ancestors are noncarers but have descendents with both male care and female care (e.g., node 2397, Figure S1), the Bayesian analysis is able to assume independent origins of female care and male care, whereas the ML analysis is forced to propose multiple scenarios concerning their order of precedence. Thus, although a “discrete” model of evolution was favored by the Bayesian analysis, the breadth of possibilities in the 1000 random scenarios generated in the ML analyses meant that a discrete model was rejected in favor of a “full” model. However, despite this uncertainty, both analyses decisively rejected the enhanced fecundity hypothesis for the evolution of male parental care and could discern broad patterns that enhance our understanding of parental care evolution in insects.

Insects exhibit all permutations of caring sexes, but transitions were nevertheless overwhelmingly between no care and female care. Insects are thus unique compared to the taxa studied to date, which are all vertebrates (see Reynolds et al. 2002; Klug et al. 2013a,b for reviews). Amphibians offer the closest comparison: like insects, transitions from noncaring ancestors were predominantly either to female care or male care. However, male care arose much more frequently in amphibians than we found for insects (Beck 1998; Reynolds et al. 2002), and biparental care arose from all other kinds of care, whereas in insects, male care hardly ever gave rise to biparental care. Other taxa show radically different patterns: in fish, for example, the predominant transition is from ancestral no care to male care (Reynolds et al. 2002; Mank et al. 2005), but less often to female care; no care can also evolve directly to biparental care and subsequently to female care (in, e.g., cichlids; Goodwin et al. 1998). Mammals show only rare transitions between female care and biparental care; reptiles show rare transitions between no care and female care with two to three transitions to biparental care (Reynolds et al. 2002). In birds, it is debated whether male care was ancestral (Varrichio et al. 2008) and thereafter gave rise to biparental care and then female care (Vehrencamp 2000) or whether female care was ancestral and independently evolved into male care and biparental care in separate lineages (Burley and Johnson 2002). In shorebirds, male care evolved into biparental care and also directly to female care (both transitions absent in insects), and biparental care evolved independently into female care or male care (Szekely and Reynolds 1995; Reynolds et al. 2002).

Why should insects show such unique transitions? As a hypothesis for future research, widespread sperm competition in insects, due partly to their spermatheca (Simmons 2001), may generally select against male involvement in care (Queller 1997; Kokko and Jennions 2008), explaining the rarity of transitions from no care to male care in insects. Second, however, sperm competition also often selects for specific male strategies to ensure paternity such as extended mate guarding, a factor that associates males with eggs and facilitates male care (Zeh and Smith 1985), helping to explain its occasional occurrence.

### EVOLUTION OF EXCLUSIVE MALE CARE: “ENHANCED FECUNDITY” VERSUS “OVERLAPPING BROODS”

There was no evidence that male care evolves out of female or biparental care, required by the “enhanced fecundity” hypothesis (Tallamy 1994, 2000). In at least one clade, *Rhinocoris* assassin bugs, the direct ancestor of the male carers is unknown and could possibly have been a female carer (*R. carmelita*), although preliminary phylogenies suggest not (J. D. J. Gilbert, unpubl. data). In most other cases, male care arose in clades without any female carers that might provide putative ancestors. Tallamy (2000) based some of his argument on observations of “male parental care” in *Hoplothrips*, *Sporothrips*, and *Idolothrips* (Crespi 1986, 1988 and unpubl. data). Here, we regard *Sporothrips* and *Idolothrips* as exhibiting female-defense polygyny rather than male care (following Costa 2006). Tallamy suggested this behavior arose out of a female-guards-eggs-male-guards-female scenario such as in *Elaphrothrips* (thus directly positing a female ancestor for male care). The thrips phylogeny, currently tentative (Mound and Morris 2007; L. A. Mound, pers. comm., although see Buckman et al. 2013) suggests this may apply for *Hoplothrips*, whose immediate relatives include female carers, but is less likely for *Sporothrips* and *Idolothrips*.

Even if we regarded these cases as male care, they would not conform to Tallamy's (1994) scenario whereby male care evolves predictably in iteroparous, hunting species where care constrains foraging, allowing caring males to “free” females to hunt and increase their fecundity. These thrips are fungus-feeders (Mound 1989). Further ill-fitting cases include male-caring Edessine bugs (Pentatomidae; Requena et al. 2010), which are almost certainly phytophagous like other Edessines (e.g., Silva and Oliveira 2010) and the majority of the Pentatomidae, and whose clade contains no known female or biparental carers. We conclude that “enhanced fecundity” is unlikely to drive initial evolution of male care. However, it is possible (indeed likely) that enhanced fecundity may have two important evolutionary effects. First, once male care has evolved, the evolution of female preference for caring males would help maintain male care, as caring males are both preferred by females and have higher offspring survival (e.g., Thomas and Manica 2005; Gilbert et al. 2010; Trumbo 2012). Second, enhanced fecundity may be a key driver of the evolution and maintenance of biparental care, discussed below.

In our sample of insects, male care arose out of no care, supporting the N2M prediction. This is consistent with the alternative proposed hypothesis for arthropods: the “overlapping broods” model (Williams 1975; Manica and Johnstone 2004) that male care can evolve where males are able to care while still being able to mate with females, thus obtaining multiple broods. In fish, where this scenario is common, external fertilization might facilitate such a transition (Gross and Shine 1981; Mank et al. 2005). In insects, which are all internal fertilizers, mode of fertilization is not relevant. However, one factor common to both insects and fish is the comparative rarity of food provisioning (and its high costs for the parent), as opposed to simple, low-cost egg guarding (Perrone and Zaret 1979). Costs and benefits of provisioning are fundamentally different compared to simply guarding (Gardner and Smiseth 2010) and the evolution of provisioning in insects drastically changes patterns of reproductive allocation compared to noncaring and guarding strategies (Gilbert and Manica 2010). Accordingly, exclusive male care occurs only among nonprovisioning species (Zeh and Smith 1985) suggesting that relatively low energetic care costs (in combination with low promiscuity costs) may be important at least in the initial evolution of male care (see Zeh and Smith 1985; Tallamy 2001; Reynolds et al. 2002; Manica and Johnstone 2004; Klug et al. 2013a,b).

Reversals from male care to no care are almost unknown (Reynolds et al. 2002), so the apparent instability of male care in hemimetabolous insects was potentially interesting. But this result must be treated cautiously until phylogenies are better resolved. A relatively high proportion of male carers occurred in groups whose phylogenies are currently unresolved and were included as polytomies (e.g., *Lopadusa*, *Edessa*, *Hoplothrips*, *Scolopocerus*). If a single male-caring species occurs in an unresolved, ancestrally noncaring clade, there is clearly one unambiguous transition from no care to male care. However, whether male care reverts back to no care depends upon the randomly resolved topology of the clade. Aggregated across trees, this reversal will be reconstructed with a high degree of uncertainty. We note also that there are no known reversals from male care in the Belostomatidae (giant water bugs, Smith 1997) or in Pycnogonids (sea spiders, Bain and Goveditch 2004), an order of arthropods related to insects—two large, speciose and exclusively male-caring groups. Three male-caring species of assassin bug (*Rhinocoris* spp.) that are morphologically almost identical probably also form a monophyletic clade (*R. tristis*, *R. albopilosus*, *R. albopunctatus*; Gilbert et al. 2010).

### EVOLUTION OF BIPARENTAL CARE: THE “MALE-JOINS” MODEL

There was broad but qualified support for the MALEJOINS prediction that biparental care arises only out of female care—the current foremost theory in arthropods (e.g., Zeh and Smith 1985; Trumbo 2012) and internally fertilizing organisms generally (Williams 1975; Dawkins and Carlisle 1976; Gross and Shine 1981). In Hemimetabola, most clades appeared consistent with the MALEJOINS assumption (Figure S1). Our Bayesian analysis of the single tree strongly supported the MALEJOINS model (Table 1B), but the ML analysis across randomly resolved trees provided less support, probably due to current uncertainty in the phylogeny of the Phlaeothripine thrips—a group containing all four parental care states, but with a basal polytomy (Figure S1) that our ML analyses randomly resolved into a range of scenarios. Generally, males are predicted to care alongside females when the benefit from doing so outweighs the benefits of deserting to seek additional mates (Maynard Smith 1977; Westneat and Sherman 1993; Queller 1997; Kokko and Jennions 2008). This might be true in cases where two parents are better than one at improving offspring survival, for example, (1) where division of labor is necessary, such as in *Trypoxylon* wasps (Coville and Coville 1980; Coville and Griswold 1984); or (2) through an “enhanced fecundity” mechanism whereby male presence allows the production of more eggs or a second clutch by females that originally cared for offspring (e.g., stomatopod crustaceans; Wright 2013). An alternative scenario is where further successful breeding by males is unlikely, such as in (1) functionally semelparous breeders such as *Cryptocercus* woodroaches (Nalepa and Bell 1997), or (2) species where costs to males of searching for additional females are prohibitive, such as *Hemilepistus* isopods (Shachak 1980). Many of these ecological conditions are met simultaneously in species breeding on rare, defensible “bonanza” resources such as burying beetles; in this group, biparental care appears to have evolved directly from no care, suggesting that intermediate stages are unstable (see Figure S1 and Eggert and Muller 1997). In contrast, a “female-joins” model has never been suggested for arthropods; this scenario was proposed for externally fertilizing taxa (e.g., Gross and Sargent 1985; Weygoldt 1987), with some empirical support in frogs (Summers et al. 1999) although not in fish (Goodwin et al. 1998; Mank et al. 2005). The male care to biparental care transition occurs in a few frogs where females return to male-guarded tadpoles and provide them with trophic eggs (Summers and Earn 1999; Brown et al. 2010), and in some clades of shorebirds as part of a general trend of evolutionary reduction of ancestral male care (Szekely and Reynolds 1995).

### PARENTAL CARE EVOLUTION IN HEMI- VERSUS HOLOMETABOLA

There were notable similarities between the Hemi- and Holometabolan patterns: for example, female care was equally labile in both groups. However, there were also important differences, particularly with regard to biparental care. Biparental care appeared reasonably stable in the Hemimetabola with low estimated rates of reversal, but when reversals occurred, they happened more often straight to no care. In contrast, in the Holometabola, transitions away from biparental care were equally likely as those towards it, and were predominantly to female care.

Biparental species in the Hemimetabola are few, comprising (1) two distinct lineages of cockroaches, the monophyletic genera *Salganea* (Maekawa et al. 1999) and *Cryptocercus*, the latter giving rise to termites (Inward et al. 2007), a hugely successful eusocial group; and (2) three species of thrips, with probably independent origins: one Phlaeothripine, *Suocerathrips linguis* (Mound and Marullo 1994), and two Idolothripines: *Bactridothrips brevitubus* (Haga 1980) and *Anactinothrips gustaviae* (Kiester and Strates 1984). The apparent stability of biparental care probably has different explanations in these different lineages. Both cockroach lineages feed on nutrient-poor wood; male and female adults feed and defend offspring with no apparent division of labor; in at least one *Salganea*, offspring receive stomodeal fluid from parents (Maekawa et al. 2008). Semelparity may select for care in *Cryptocercus* (Tallamy and Brown 1999; Trumbo 2012), although *Salganea* are probably iteroparous (Maekawa et al. 2008). As labor is not clearly divided, lack of outside options for males may select for paternal investment, as for *Hemilepistus* isopods (Shachak 1980) rather than factors such as defensibility of the nest (see Trumbo 2012 for discussion). Offspring are altricial, reflecting reduced investment in offspring structures such as cuticle and eyes as parental care intensified (Nalepa et al. 2008; Nalepa 2011). Such reduction may effectively lock the lineage into intensive parental care, whereas in other lineages of Hemimetabola, and even in closely related cockroaches, offspring feed relatively independently. In contrast, the three thrips species are relatively new discoveries; they may appear as singletons within their respective clades because their immediate relatives are poorly known along with this group in general, reducing the detectability of any evolutionary reversals of biparental care.

In the Holometabola, larvae are already generally soft-bodied, giving less potential for any further reduction of these structures that might trap any particular lineage into a strategy of care. Again, biparental care is not noticeably associated with semelparity, appearing in lineages both functionally semelparous (e.g., burying beetles) and highly iteroparous (e.g., Passalidae). Biparental care appears most extensively in beetles—primarily in the Passalidae, Scarabaeidae, Scolytidae, and in burying beetles, with isolated exceptions (e.g., the Tenebrionid *Parastizopus armaticeps* [Rasa 1990], cossonine weevils *Araucarius* spp. [Kirkendall et al. 1997]). Biparental care has also evolved in the solitary crabronine Hymenoptera (e.g., *Trypoxylon* spp. [Coville and Coville 1980; Coville and Griswold 1984]). Valuable, defensible nests or resources that specifically select for division of labor are a consistent feature of these systems, with one parent, typically the male, focusing on defense whereas the other focuses on nest construction and/or provisioning (Eickwort 1981; Trumbo 2012). The apparent lability of biparental care in Holometabola appears to be due primarily to cases in the Scolytidae, with at least five reversals to uniparental female care (see Figure S1), whereas another reversal is possible in the Cossoninae. Why selection for male involvement in care should be weaker or more variable in weevil lineages than in other beetle clades requires further study, but may be related to their specific ecology.

Taken together, these patterns suggest two hypotheses: first, “nest-based” selection for biparental care, that is, arising from a nest constituting a central place that requires both foraging and defense, thus selecting for division of labor, may be less evolutionarily stable than selection arising from semelparity or from lack of outside options for males. Second, this nest-based selection may be commoner in the Holometabola than in Hemimetabola, owing to the former having highly altricial offspring.

### FUTURE DIRECTIONS

Given the inevitable uncertainty of the insect phylogeny, the degree and robustness of support our findings provide for existing theory is encouraging. Future research should aim to integrate these findings with comparative data on, for example, life history trade-offs (e.g., Berrigan 1991; Honěk 1993; Gilbert and Manica 2010), social environments (Smiseth and Moore 2004; Wong et al. 2013), geography (Purcell 2011), and food provisioning (Gilbert and Manica 2010). The existing wealth of comparative field data (e.g., Tallamy and Denno 1981; Requena et al. 2009; Gilbert et al. 2010; Hanelová and Vilímová 2013) provides a rich source of material for such studies. Our findings should also provide an initial framework in which to test theoretical models (e.g., Field and Brace 2004; Field 2005; Bonsall and Klug 2011; Gardner and Smiseth 2010; Klug et al. 2013a,b). Two key hypotheses are (1) the semelparity hypothesis, i.e. that semelparity should favor female care (Tallamy and Brown 1999), which has received mixed support (Stegmann and Linsenmair 2002; Trumbo 2013) and (2) the hypothesis that female care in the Hemiptera is a costly relic whose distribution is explained better by multiple losses than by multiple gains (Tallamy and Schaefer 1997), again receiving mixed support (Lin et al. 2004). Finally, ecological drivers of parental care evolution also clearly warrant attention. More than 40 years after Wilson proposed his four “prime movers” of insect parental care (Wilson 1975), despite being widely discussed and reviewed (e.g., Tallamy and Wood 1986; Costa 2006; Trumbo 2012), and recently modeled (Bonsall and Klug 2011; Klug et al. 2013a,b) neither Wilson's prime movers nor Tallamy and Wood's (1986) “resource hypothesis” has been rigorously tested.

## Supporting Information

Disclaimer: Supplementary materials have been peer-reviewed but not copyedited.

**Figure S1**. Full tree used in this study (2013 tips).

**Table S1**. References used for assembling phylogeny of insects.

**Table S2**. Table of calculations for the number of models in the universe of possible models where the number of zero parameters > 0 (after Currie et al. 2010).

**Table S3**. Full dataset used in this study, provided in MS Excel (.xslx) format. Key to care states: N, no care. F, female-only care. M, male-only care. B, biparental care.

**Table S4**. Full dataset used in this study, provided in comma-delimited (.csv) format. Key to care states: N, no care. F, female-only care. M, male-only care. B, biparental care.
