# Supplementary material for: The evolution of parental care in insects: A test of current hypotheses
Source: Evolution. 2015 Apr 30;69(5):1255–70. doi: 10.1111/evo.12656 (PMC4529740; doi:10.1111/evo.12656)
Supplement: Supplementary file 1 — Figure S1. Full tree used in this study (2013 tips). [file evo0069-1255-sd1.pdf]

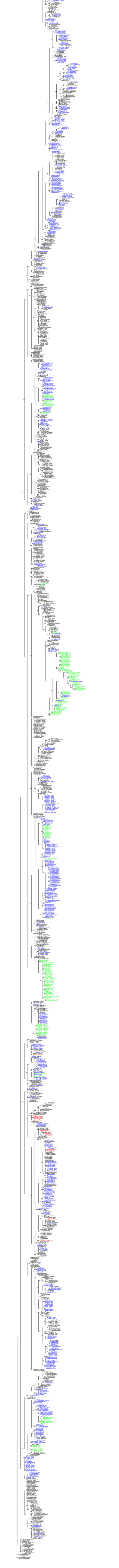

Figure S1. Full tree used in this study (2013 tips). Key to care states: black = no care; blue = female care; red = male care; green = biparental care. For sources used to assemble this tree, see Table S1.
